# Descriptive study of onychomycosis in a hospital in São Paulo

**DOI:** 10.1590/S1517-838246220130541

**Published:** 2015-06-01

**Authors:** Clarissa Santos de Carvalho Ribeiro, Clarisse Zaitz, Valéria Maria de Souza Framil, Thaíssa Santos de Carvalho Ottoboni, Melissa Santos de Carvalho Tonoli, Renata Pinheiro Ribeiro

**Affiliations:** 1 Faculdade de Ciências Médicas da Santa Casa São Paulo, Departamento de Dermatologia, Faculdade de Ciências Médicas, Santa Casa de São Paulo, São Paulo, SP, Brasil, Departamento de Dermatologia, Faculdade de Ciências Médicas, Santa Casa de São Paulo, São Paulo, SP, Brazil.; 2 Faculdade de Medicina de Itajubá, Faculdade de Medicina de Itajubá, Itajubá, MG, Brasil, Faculdade de Medicina de Itajubá, Itajubá, MG, Brazil.; 3 Universidade Estadual Paulista, Universidade Estadual Paulista, São José dos Campos, SP, Brasil, Universidade Estadual Paulista, São José dos Campos, SP, Brazil.; 4 Universidade Federal de Alfenas, Universidade Federal de Alfenas, Alfenas, MG, Brasil, Universidade Federal de Alfenas, Alfenas, MG, Brazil.

**Keywords:** onychomycosis, dermatophytes, yeasts, non-dermatophytes fungi, *Prothoteca* spp

## Abstract

Onychomychosis, a nail fungus infection is the most frequent nail ailment, constituting about half of all nail disorders. It can be caused by dermatophytes, non-dermatophytes, yeasts and *Prothoteca* spp. Methods include 5407 samples of patients with suspected onychomycosis, studied from January 2002 to December 2006, by direct mycological examination and fungi culture. The diagnosis of onychomycosis was confirmed in samples from 3822 direct mycological and/or culture positive. The diagnosis was established by culture for fungi. Among the 1.428 identified agents, the dermatophytes were responsible for 68.6% (N = 980) of cases, followed by yeasts with 27.6% (N = 394), non-dermatophytes fungi with 2.2% (N = 31), *Prothoteca* spp with 0.1% (N = 2), and associations with 1.5% (N = 22). Females were more affected, with 66% (N = 2527) of cases, and the most affected age group ranged from 31 to 60 years of age (median 47 years). Fungal microbiota is often changed in the world, both quantitatively and qualitatively, and is affected by several environmental factors. Thus, the periodic review of the composition of this microbiota is important to evaluate the epidemiology and thus proportion a better therapeutic response.

## Introduction

Onychomycosis is a fungal nail infection which can be caused by dermatophytes, yeast, non-dermatophyte filamentous fungi (NDFF) and currently *Prototheca* spp, affecting approximately 8% of the world population (Effendy I *et al.*, 2005; Zaitz C *et al.*, 2006).

This nail ailment represents 30% of the fungal surface and around 50% of nail diseases (Ghannoum MA *et al.*, 2000). The main onychomycosis etiologic agents are dermatophytes, which are isolated in 75% of cases. Candida yeasts occur in around 18–20% of cases and NDFF reach percentages ranging from one to five percent of cases (Agarwalla A *et al.*, 2006).

Epidemiological studies of onychomycosis can provide divergent results in the literature due to several factors, such as environmental factors and related to the host ones. Key environmental factors considered include: urban development, industrialization, geographical location and climatic conditions such as temperature and exposure to ultraviolet rays. Those factors related to the host are cited in the literature as being: age, lifestyle, occupation, sex, color, and chronic diseases (Sigurgeirsson B *et al.*, 2000).

Onychomycosis is considered a public health problem due to high prevalence associated to morbidities such as diabetes, impaired peripheral circulation, ungueal trauma repetition, immunodeficiencies (Elewski BE, 1998). It is a disease which does not demand compulsory notification, thus making it difficult to access the extent of the problem in our midst. Thus, onychomycosis affects the quality of a patient's life and causes esthetic losses, reflecting directly on self-esteem, vanity, social discrimination and the patient's working potential (Lopes JO *et al.*, 1999).

Although nail infections are common, nowadays they are associated with difficult treatment and are reported as having high rates of recurrence and therapeutic inefficacy. The knowledge of ecology, etiology and distribution of the biota of the main etiological agents allows for a better comprehension of natural history, evolution and may contribute in the future for new therapeutic modalities (Schroeff J *et al.*, 1992).

In this retrospective study, an analysis of all positive mycological examinations from patients with clinical onychomycosis diagnosis from the São Paulo Hospital Dermatology Department's Mycology Laboratory was performed, covering a period from January 2002 to December 2006

The objectives were: 1) To analyze the sensitivity of a diagnostic laboratory for onychomycosis in the São Paulo Hospital Dermatology Department's Medical Mycology Laboratory. 2) To analyze the distribution of cases of onychomycosis in relation to demographic data: age and sex. 3) To analyze the distribution of onychomycosis cases according to the main etiological agents: dermatophytes, non dermatophyte filamentous fungi (NDFF), protists and yeasts.

## Material and Methods

This paper includes a descriptive study conducted at the São Paulo Hospital Dermatology Department, by reviewing the results of mycological examinations performed from January 2002 to December 2006.

All patients seen at the dermatology ambulatory that presented, among the possible diagnoses, a clinical suspicion of onychomycosis, were submitted to mycological examination in order to confirm the onychomycosis diagnosis.

Reagents, culture media, equipment and consumable material are routinely used in the medical mycology laboratory of the Dermatology Department in Hospital in São Paulo. The study was approved by the Ethics and Research Committee of the São Paulo Hospital.

### Laboratory procedures

#### Direct microscopic examination

The material was obtained by scraping with a dental explorer, a dental curette nail and examined between a slide and coverslip after clarification with potassium hydroxide with 20% dimethyl sulphoxide added (Lacaz CS *et al.*, 2002; Sidrim JJC and Rocha MFG, 2004).

#### Culture

Macroscopic aspects: The culture media for isolation and analysis of morphological species of Dermatophytes are Sabouraud's medium with chloramphenicol and Sabouraud medium with cycloheximide. Non-dermatophyte filamentous fungi, yeast and *Prototheca* spp are grown on Sabouraud cloranfenicol. In our laboratory, the material was inoculated into three tubes of the means mentioned: first, collection of clinical material and isolation of agents; second, collection of clinical material after the first seven days from the initial collection and isolation of the agent; the third collection of clinical material after seven days from the second collection and isolation of agents. The growth period to occur during the full maturity of fungal structures is: dermatophytes and NDFF observed around 15 days, *Prototheca* spp and yeast about seven days (Lacaz CS *et al.*, 2002; Sidrim JJC and Rocha MFG, 2004).

Microscopic aspects: The technique of microculture on slides with a culture medium with agar-cornmeal for the identification and determination of genus and species of dermatophytes, yeasts and NDFF was used. In *Prototheca* spp, an inoculum of the culture was withdrawn to be identified and was placed on a slide with lacto-phenol blue dye cotton. It was possible to observe the shaped structures of morula. Species identification is performed through biochemical tests. (Lacaz CS *et al.*, 2002; Sidrim JJC and Rocha MFG, 2004).

### Statistical analysis

To analyze the results, the chi-square test in MINITAB 14 and tables in EXCEL 2007 were used for characterization of the Clinical sample material obtained from nails of patients with suspected onychomycosis. This was done to evaluate the association between the variables of age, gender, test result, the affected site and agent. In all tests, the 0.05 significance level was set.

## Results

### Characterization of the samples studied

#### Direct microscopic examination and culture

Between January 2002 and December 2006, 5407 mycological tests were carried out with clinical suspicion of onychomycosis in 3541 patients of any age, sex and race, which were analyzed by direct microscopic examination and/or culture. Of the total amount tested, 71% (3.822/5.407) were positive for laboratory diagnosis of onychomycosis. Of the remaining mycological examinations that were analyzed, 29% (1585/5407) were negative.

**Figure 1:**
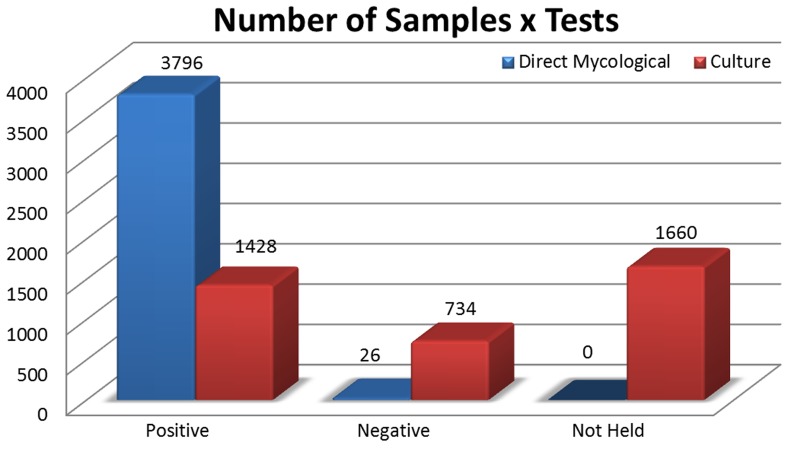
Distribution of 3822 direct microscopic examination and cultures in patients with clinical diagnosis of onychomycosis in the Mycology Laboratory of the São Paulo Hospital Clinical Dermatology Department between January 2002 and December 2006.

The distribution of mycological examinations analyzed (Figure 1) was based on laboratory confirmed cases of onychomycosis using direct microscopic examination and culture (both are criteria to define positivity).

#### Onychomycosis age and gender

Regarding age, it was observed in the table below that among the 3822 samples with a diagnosis of onychomycosis, 60% (2.287/3.822) presented data on age, with a minimum age of three months (0.25 years) and the maximum age of 98 years. The mean age was 46.9 years, standard deviation ± 17.02; observing a coefficient of variation of 36.3%, concluding that the most representative of the age of this population would be the median: 47 years old.

**Figure 2:**
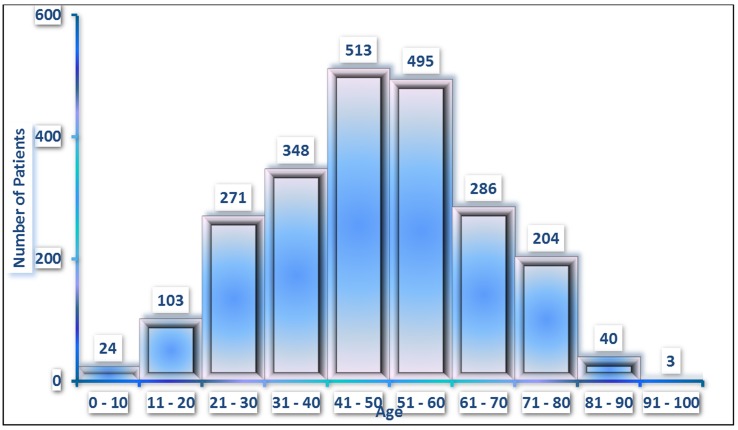
Age distribution of patient samples with clinical and laboratory of onychomycosis in the Mycology Laboratory of the Dermatology Department the São Paulo Hospital between January 2002 and December 2006.

The total distribution of patient samples analyzed in relation to age is shown in Figure 2. Most samples of patients (60%) are aged from 31 to 60 years.

In terms of the distribution in relation to sex of the 3822 mycological positive tests for laboratory diagnosis of onychomycosis, 2527 patients were female (66%) and 1295 were male (34%).

Table 1 shows the distribution according to age and sex in cases of onychomycosis.

**Table 1:** Distribution of samples from patients with clinical and laboratory of onychomycosis in the mycology laboratory of the Clinic Dermatology, at the São Paulo Hospital in the period between January 2002 and December 2006, regarding both age and sex.

Age	Positive results		Total	%
			
	Female	Male		
0–10	8	16	24	1.05%
11–20	65	38	103	4.50%
21–30	165	106	271	11.85%
31–40	207	141	348	15.22%
41–50	391	122	513	22.43%
51–60	345	150	495	21.64%
61–70	196	90	286	12.51%
71–80	124	80	204	8.92%
81–90	34	6	40	1.75%
91–100	3		3	0.13%
Total	1538	749	2287	100%

p < 0.0001 p < 0.05 was considered significant.

The chi-square statistical test, performed to evaluate the association between the positive result of the test variables and age of the patients by sex, showed a significant difference (p < 0.0001) between the percentage of positive results observed in relation to patients age by sex, for the level of significance; that is, the highest incidence of positive results was in the range of 31 to 60 years for females.

#### Identification of the etiologic agents of onychomycosis

**Figure 3:**
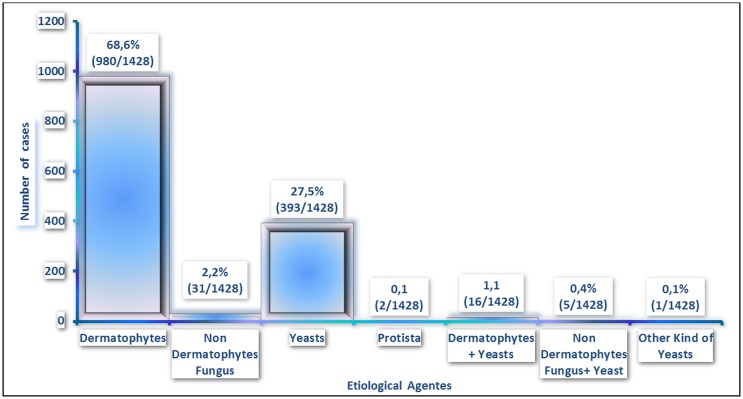
Distribution of groups of etiologic agents isolated from samples of patients with onychomycosis at the Dermatology Department of São Paulo Hospital between January 2002 and December 2006.

The percentage of culture isolation and identification of agents etiology was 66% (1.428/2.162) of the samples. The distribution was as follows: to 68.6% dermatophytes (980/1.428) NDFF with 2.2% (31/1.428), yeast with 27.5% (393/1.428), *Prototheca* spp 0.1% (2/1.428) and associations between the etiologic agents such as dermatophyte + yeast 1.1% (16/1.428), yeast + NDFF 0.4% (5/1.428), other kind of yeasts 0.1% (1/1428) (Figure 3).

Table 2 shows the main etiological agents in relation to gender and species, in samples from patients with onychomycosis. The most common dermatophyte, *Trichophyton rubrum*, registered 55.7% (796/1.428); the most common NDFF was *Fusarium* spp with 1.6% (23/1.428). The yeast *Candida* spp was observed with 27.2% (388/1.428) and *Prototheca* spp 0.1% (2/1.428).

**Table 2:** Distribution of genera and species isolated from samples of patients with onychomycosis at the Dermatology Department of the São Paulo Hospital between January 2002 and December 2006.

Etiological agent	Number of samples	Percentage
Dermatophyte	980	68.6%
*Epidermophyton floccosum*	2	0.1%
*Microsporum gypseum*	3	0.2%
*Trichophyton raubistscheckii*	1	0.1%
*Trichophyton mentagrophytes*	97	6.8%
*Trichophyton rubrum*	796	55.7%
*Trichophyton* spp	45	3.2%
*Trichophyton tonsurans*	36	2.5%
Dermatophyte + Yeasts	16	1.1%
*Trichophyton tonsurans* e *Candida* spp	5	0.4%
*Trichophyton mentagrophytes* e *Candida* spp	1	0.1%
*Trichophyton rubrum* e *Candida* spp	9	0.6%
*Trichophyton* spp e *Candida* spp	1	0.1%
NDFF	31	2.2%
*Acremonium* spp	1	0.1%
*Aspergillus* spp	1	0.1%
*Fusarium* spp	23	1.6%
*Scytalidium hyalinum*	6	0.4%
NDFF + Yeasts	5	0.4%
*Fusarium* spp e *Candida* spp	5	0.4%
Yeasts	394	27.6%
*Candida* spp	388	27.2%
*Trichosporon* spp	5	0.4%
*Trichosporon* spp e *Candida* spp	1	0.1%
Protista	2	0.1%
*Prototheca* spp	2	0.1%
Total	1428	100.0%

p < 0.0001 p < 0.05 was considered significant.

The chi-square statistical test, conducted to evaluate the association between the positive result variables of the examination and the type of etiologic agent, showed a difference (p < 0.0001) between the percentage of positive results found in the type of etiologic agent for the level of significance, that is, a higher positive incidence refers to dermatophytes.

## Discussion

Onychomycosis epidemiology has multifactorial influence and its prevalence is directly related to age and other population factors, such as lifestyle and association with other diseases. Furthermore, the distribution of pathogens, agents of onychomycosis is not uniform, depending on several factors such as geography, climate of the region, and population migration (Faergemann J, Baran R, 2003; Brito A, 1992).

In the São Paulo Hospital Dermatology Department's Mycology Laboratory, from January 2002 to December 2006, 5407 patients with clinical suspicion of onychomycosis were evaluated. The laboratory diagnosis (mycological and/or culture positive) was confirmed in 71% (3.822/5.407) of the samples analyzed. Elewski BE *et al.* in 1997 observed that, in 2065 clinical samples with suspected onychomycosis, 82% were diagnosed cases (1.707/2.065). Gupta *et al.*, in a multicenter study in 2000, found that of the 15.000 patients who had nails with some sort of abnormality, only 8% of cases (1.199/15.000) onychomycosis were confirmed by laboratory tests. In a study carried out by Brilhante *et al.* in 2005, of the 976 patients with suspected onychomycosis, 52% (512/976) were confirmed as having the disease. In Belgium, 36.04% of tests confirmed presence of onychomysis in the studied patients (1.290/3.579) (Arrese JE *et al.*, 2005). A study conducted in India shows that of the 302 patients, 42.4% (128/302) had the diagnosis confirmed by laboratory examination (Sarma S *et al.*, 2008).

Of the 3822 clinical samples with direct examination and/or positive cultures analyzed in our study, the direct microscopic examination was positive in 99.3% (3.796/3.822) of them, while the culture was positive in 66% (1.428/2.162) of the total samples. Studies in the literature reported 82.45% (94/114) positivity for the direct microscopic examination, and 17.5% (20/114) for culture (Brito A, 1992). The author explains the difference was due to the difficulties that the mycology laboratory went through some time, resulting in damage to research. In 1997, a positivity of 82% (1.707/2.065) for direct microscopic examination and 52% (1.069/2.065) for culture was observed (Elewski BE *et al.*, 1997).

Culture was performed in 56.6% (2.162/3.822) of all samples studied; it was positive in 66% (1.428/2.162). In the studied laboratory, due to the large number of surveys collected in the daily routine, all positive culture direct mycological examination are not made. The cultivation limitations of all samples are the cost of the test and the period necessary for fungus growth.

Comparing our results with the literature, it can be stated that the studied laboratory is efficiently achieving results similar to the Mycological reference laboratories. Besides the difficulty related to culture in all samples studied, it was observed that the positivity obtained by direct examination is significantly higher than that one obtained by culture. This fact can be explained by the uneven distribution of fungi in lesions, the difficulty to collect material properly (especially in the subungueal region) and the ease by which the contamination by airborne fungi and bacterial microbiota, thus hindering the identification of the true etiologic agent, due to limitations inherent in the examination, such as the intense keratinization of nails, which makes microscopic observation of microorganisms becomes difficult; for fungal viability, which can result in false negative cultures and the use of antifungal medications by the patient prior to collection (Carvalho MTF, 1990; Martelozzo IC *et al.*, 2005).

The age group with confirmed onychomycosis ranged from three months to 98 years of age, with a median age of 47 years, the age group 31–60 years, considered the economically active population, was involved in 60% of our sample. Our results are consistent with those of Martins *et al.*, in 2007, who observed in a study with 184 patients, the mean age of onset was 36 to 64 years (62%). Already, in a study with 302 patients, it was found that onychomycosis occurred more in the age group between 21 and 30 years (36%) (Sarma S, 2008).

Of the 3822 positive mycological examinations for laboratory diagnosis of onychomycosis, 2527 were from female patients (66%) and 1295 male (34%).

According to statistical analysis by chi-square test, there is a significant difference between positive tests in both sexes. These data are consistent with the literature, as some authors have observed that the frequency of onychomycosis ranging from 67% to 74% in females (Souza EAF *et al.*, 2007; Effendy I *et al.*, 2005; Alvarez MI *et al.*, 2005; Koussidou T *et al.*, 2002; Vélez A *et al.*, 1997; Kemna ME and Elewski BE, 1996; Sais G *et al.*, 1995; Schroeff J *et al.*, 1992). Other authors found exactly the opposite, with about 64% of cases occurring in males (Sarma S *et al.*, 2008; Ghannoum MA *et al.*, 2000, Sigurgeirsson B and Steingrimsson O, 2000; Perea S *et al.*, 2000). The reason for these discrepancies may lie in the composition of the population studied, since the fungal infection depends on cultural habits and ecology, as previously described.

Similarly the sample shows the predominance of female patients. In this sampling, the most affected age group was between 31 and 60 years, where greater and more significant (p < 0.0001) incidence of positive results was seen in the range of 31 to 60 years for females. In the literature, studies like those of Ghannoum *et al.* in 2000 reported a higher incidence of onychomycosis in the active age group with a mean age of 57 years mainly in males (58%) (1063/1832). Patients with onychomycosis were in the range of 40–50 years old (67.4%) of which 71% were female (179/252) (Martins EA *et al.*, 2007; Martelozzo IC *et al.*, 2005).

Some authors explain the increased prevalence of onychomycosis with aging due to some factors, such as peripheral circulation slower, inactivity, inability to cut and care for nails, presence of comorbidities (diabetes, repeated nail trauma, longer exposure to pathogenic fungi, lower immunity). Moreover, the reason why the prevalence of onychomycosis was lower in children, can be justified by quicker nail growth, less exposure to the etiologic agents, a lower prevalence of tinea pedis and to a lesser extent nail invasion (Tosti A *et al.*, 2005; Elewski BE, 1998).

Dermatophytes are the etiological agents responsible for most onychomycosis, representing approximately 75% of these infections (Afsaneh AMD *et al.*, 2008; Seebacher CJ *et al.*, 2007; Summerbell RC, 2005; Araújo A *et al.*, 2003; Rodriguez JMT and Jodra OL, 2000; Gupta AK *et al.*, 2000; Weitzman I and Summerbell RC, 1995; Gupta AK, 1997). Some authors found different proportions: 33.85% and 41% for dermatophytes; 13.97% and 13% for non-dermatophyte filamentous fungi and 52.17% and 46% for yeast, respectively (Martelozzo *et al.*, 2005; Kemna *et al.*, 1996). There are few reports of onychomycosis "simile" caused by *Prototheca* spp (Zaitz C *et al.*, 2006; Magerman K, 1991; Marcano C and Feo M, 1981).

In this study, dermatophytes were the main etiologic agents isolated (68.6% 980/1.428) followed by yeast in 393 patients (27.5%), with the NDFF (2.2%); the two *Prototheca* spp 0.1% cases, and finally etiological agents associations with 1.6% (22/1428). It was observed that when the dermatophytes species was analyzed, the *T. rubrum* was isolated 81.2% (796/980) of the time, followed by *T. mentagrophytes* in 9.9% (97/980), *T. tonsurans* in 3.6% (36/980), *M. gypseum* in 0.2% (3/980), *E. floccosum* in 0.1% (2/980) and *T. raubistscheckii* in 0.1% (1/980).

On reviewing the literature, it was found that the main agents of onychomycosis are: *T. rubrum*, *T. mentagrophytes* and *T. tonsurans* (Ghannoum MA *et al.*, 2000; Rodriguez JMT and Jodra OL, 2000; Brito A, 1992). *T. raubistscheckii* is considered by many mycologists a variant of the *T. rubrum* and it is rarely isolated in the nail (Brasch J, 2007, Papini M *et al.*, 2004).

Furthermore, non-dermatophyte filamentous fungi (NDFF) are cited in literature as etiologic agents of onychomycosis. In this study, *Fusarium* spp was the most isolated NDFF with 74.2% (23/31). In following came the *Scytalidium hyalinum* with 19.3% (6/31), *Acremonium* spp and *Aspergillus* spp, both with 3.2% (1/31).

It was found that 13.6% of onychomycosis were caused by NDFF. The main etiologic agent identified by the authors was *Fusarium* spp, 21.2% (28/132) (Brasch J, 2007).

For yeast isolates, *Candida* spp was found in 98.5% (388/394) (Souza EAF *et al.*, 2007; Martelozzo IC *et al.*, 2005). It was observed that the yeast Candida was most frequently isolated from cases of onychomycosis. The *Trichosporum beigelli* was isolated in 1.2% (5/394) of our cases as the etiological agent of onychomycosis by yeast and some studies also have similar results (Brasch, 2007; Papini M *et al.*, 2004). Associations and fungi such as onychomycosis agents were also found.

Dermatophyte and yeast occurred in 1.1% of cases (16/1.428) NDFF with yeast at 0.4% of cases (5/1.428) and yeasts from other genera (not Candida) in 0.1% of cases (1/1.428). There are few studies which mention mixed onychomycosis. Studies reported association of onychomycosis caused by *Candida* spp and other fungi in immunosuppressed patients and reported possible associations of fungi in onychomycosis in a study of 2766 patients(Koussidou T *et al.*, 2002). Onychomycosis "like" caused by *Prototheca* spp was a peculiarity observed in our study. In respect to this peculiarity, two patients were observed (patients with the same two nails affected), which correspond to 0.1% of the causative agents of onychomycosis. This case was published, the first case in Brazil of onychomycosis caused by *Prototheca* spp and 3rd case in the world (Zaitz C *et al.*, 2006).

## Conclusions

From January 2002 to December 2006, 3822 samples were analyzed with clinically suspected onychomycosis which were confirmed in the laboratory and allowed for the following conclusions:

1. The direct microscopic examination method is sensitive, rapid and inexpensive for general diagnosis of onychomycosis. In spite of its culture high specificity, it is difficult to perform, more costly and dependent on several factors such as: collection, culture medium and the skills of the professional who performs it.
2. The most affected age group in the population studied was 31 to 60 years of age and predominantly female (66%).
3. The main groups of etiologic agents were isolated: dermatophytes (68.6%), NDFF (2.2%), yeast (27.5%), *Prototheca* spp (0.1%) and associations fungi (1.6%). The distribution of species most often found in different groups of agents was: a Dermatophytes: *T. rubrum* (81.2%), *T. mentagrophytes* (9.9%) and *T. tonsurans* (3.6%), b. NDFF: *Fusarium* spp (74.2%) and *Scytalidium hyalinum* (19.3%) c. Yeasts: *Candida* spp (98.5%).
